# Assessment of Cancer‐Related Cognitive Impairments Across Anti‐Cancer Therapies in a Predominantly Breast Cancer Cohort

**DOI:** 10.1002/ijc.70691

**Published:** 2026-08-07

**Authors:** Qiqi Lei, Martina E. Schmidt, Philipp Zimmer, Patric Meyer, Andreas Schneeweiss, Karen Steindorf

**Affiliations:** ^1^ Division of Physical Activity, Cancer Prevention and Survivorship German Cancer Research Center (DKFZ) and National Center for Tumor Diseases (NCT) Heidelberg, a Partnership Between DKFZ and University Medical Center Heidelberg Heidelberg Germany; ^2^ Medical Faculty, University of Heidelberg Heidelberg Germany; ^3^ Division of Sports Medicine Institute for Sport and Sport Science, TU Dortmund University Dortmund Germany; ^4^ School of Psychology, SRH University of Applied Sciences Heidelberg Heidelberg Germany; ^5^ Department for General and Applied Linguistics Ruprecht‐Karls‐University Heidelberg Heidelberg Germany; ^6^ Network Aging Research (NAR), Ruprecht‐Karls‐University Heidelberg Heidelberg Germany; ^7^ Division of Gynecological Oncology National Center for Tumor Diseases (NCT) Heidelberg Heidelberg Germany

**Keywords:** breast cancer, cancer survivorship, cancer‐related cognitive impairment, chemotherapy, endocrine therapy

## Abstract

Cancer survivors frequently experience cancer‐related cognitive impairment (CRCI), but the contributions of different anti‐cancer therapies remain unclear. Patients with breast or gynaecological cancer were recruited before any anti‐cancer treatment. Cognitive function was assessed by objective and subjective measures pre‐treatment (T0), post‐treatment (T1), and 1 year after T1 (T2). Objective measures include Hopkins Verbal Learning Test‐Revised (HVLT‐R), Trail Making Test (TMT), Controlled Oral Word Association Test (COWA), and Mnemonic Similarity Task (MST). Subjective measures include Functional Assessment of Cancer Therapy—Cognitive scale (FACT‐Cog) and European Organization for Research and Treatment of Cancer Quality of Life Questionnaire Core 30 (EORTC QLQ‐C30). Linear mixed models were performed. Patients (*n* = 234) were of age 55.4 ± 11.6 years. At T1, chemotherapy was associated with worse visual searching and motor speed (TMT‐A: *β* = 0.36, 95% CI: 0.03–0.69), perceived global function (FACT‐Cog Global Function: *β* = −0.39, 95% CI: −0.67 to −0.11), and cognitive function (EORTC QLQ‐C30: *β* = −0.50, 95% CI: −0.82 to −0.18) compared to endocrine therapy. At T2, endocrine therapy was associated with poorer verbal learning and memory performance (HVLT‐R total recall: *β* = −0.40, 95% CI: −6 to −0.03) and higher perceived cognitive impairments (FACT‐Cog PCI: *β* = 0.34, 95% CI: 0.05–0.63). In conclusion, CRCI may occur during certain types of chemotherapy but appears to ameliorate over time. Ongoing endocrine therapy may have longer‐lasting impact on cognitive function. Appropriate counselling for informed decision making and good supportive care is needed.

## What's New?

Cancer‐related cognitive impairment (CRCI) arises from various factors, including treatment. However, the cognitive effects of different anticancer therapies remain poorly understood. In this investigation of patients with breast or gynaecological cancer, the authors assessed cognitive performance and patient‐reported cognitive function before and after treatment. Declines in cognitive performance and perceived cognitive function were associated with chemotherapy, though over time, these impairments largely improved. By comparison, endocrine therapy was linked to more persistent impairments in verbal learning, memory, and self‐reported cognition. The findings support personalised survivorship care and highlight the need to monitor cognitive outcomes according to treatment type.

## Graphical abstract

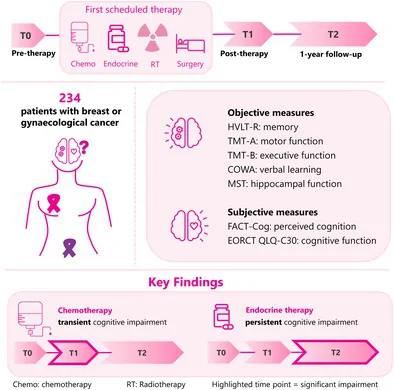

**Abbreviations**
95% CIs: 95% confidence intervals
CESD‐R: the Revised 20‐item Center for Epidemiologic Studies Depression Scale
CogniFiT: study name, Cognitive Function in patients with Tumors
COWA: Controlled Oral Word Association Test
CRCI: cancer‐related cognitive impairment
EORTC QLQ‐C30 CF: European Organization for Research and Treatment of Cancer Quality of Life Questionnaire Core 30, Cognitive Function scale
ESSI: ENRICHD Social Support Instrument
FACT‐Cog GF: Functional Assessment of Cancer Therapy—Cognitive scale, Global function
FACT‐Cog iQoL: FACT‐Cog, impact on Quality of Life
FACT‐Cog PCA: FACT‐Cog, Perceived Cognitive Abilities
FACT‐Cog PCI: FACT‐Cog, Perceived Cognitive Impairments
FDR: false discovery rate
HVLT‐R: Hopkins Verbal Learning Test‐Revised
MST LDI: Mnemonic Similarity Task Lure Discrimination Index
MST REC: Mnemonic Similarity Task Corrected Recognition Score
PSQI: Pittsburgh Sleep Quality Index
SD: standard deviation
STAI‐T: State–Trait Anxiety Inventory Test
TAILORx: Trial Assigning Individualized Treatment Options
TMT‐A: Trail Making Test, part A
TMT‐B: Trail Making Test, part B

## 1 Introduction

Advances in anti‐cancer therapies have significantly improved the overall survival of cancer patients [1], yet many patients experience persistent side effects that impair quality of life [2]. Among these side effects, cancer‐related cognitive impairments (CRCI) have been reported to affect up to 75% of cancer patients and encompass impairments in memory, attention, executive functions, language comprehension, and processing speed [3]. Patients also report subjective concerns such as forgetfulness, distractibility, and word‐finding difficulties [4]. CRCI can occur during treatment, immediately afterward, or with delayed onset [5]. Meanwhile, CRCI is recognised as a multifactorial tumour burden which has led to research into its underlying mechanisms, including anti‐cancer treatments, as well as interactions with other side effects, such as fatigue, anxiety, depressive symptoms, and sleep problems [6, 7].

Assessment of CRCI in cancer survivors is typically divided in two main aspects: objective neuropsychological tests and subjective cognitive symptoms reported by cancer survivors [8]. Objective measures refer to cognitive data gathered by standardised tests in which participants are required to perform tasks to the best of their capabilities [9]. Subjective measures refer to patients' self‐report on cognitive abilities or other symptoms that might affect quality of life [8]. Previous studies have indicated that objective tests rarely correlate with self‐reported cognitive functions [2]. Neuropsychological tests evaluate cognition at one time point while self‐reported questionnaires assess cognitive status over a previous period and across different contexts, which may contribute to low correlation [10].

It is also important to distinguish CRCI from dementia. CRCI reflects subtle cognitive changes occurring in the context of cancer and its treatment, whereas dementia is a progressive neurodegenerative syndrome. There is currently no evidence that CRCI increases the risk of dementia. In contrast, a large cohort study with Mendelian randomization analyses reported a lower incidence of dementia among individuals diagnosed with cancer compared with participants without cancer [11]. Similarly, a nationwide population‐based cohort study found that breast cancer survivors had a lower risk of developing Alzheimer's disease than age‐matched cancer‐free controls [12]. Collectively, current epidemiological evidence does not suggest an increased risk of dementia among cancer survivors.

Although definitive mechanistic evidence in humans remains limited, preclinical studies link specific chemotherapeutic agents to plausible biological pathways relevant to CRCI [13, 14]. Taxane has been associated with neuroinflammation, oxidative stress, impaired neurogenesis and altered synaptic plasticity, likely related to their microtubule‐stabilising actions and mitochondrial effects in neuronal tissues. Anthracyclines have been implicated in oxidative stress [15] and cytokine‐mediated neuronal injury, with downstream effects on neurogenesis and neurotransmitter pathways [16]. Platinum‐based drugs such as carboplatin can induce mitochondrial DNA damage, increased oxidative stress, and pro‐inflammatory signalling, which may contribute to cognitive alterations [17]. Despite these agents primarily cause chemotherapeutic‐induced peripheral neuropathy by damaging peripheral neurons that lie outside the protection of the blood–brain barrier, the oxidative responses and systemic inflammatory response they trigger can also disrupt this barrier and contribute indirectly to central neurotoxicity and CRCI [18, 19].

Beyond chemotherapy, emerging evidence indicates that other anti‐cancer treatments, such as endocrine therapy and targeted therapy, can also induce cognitive deficits [3, 20]. The changing landscape of cancer treatments and the increasing number of long‐term survivors have drawn more attention to CRCI. It has also been hypothesised that cancer and its treatments can cause sufficient damage to accelerate aging and cognitive declines [21, 22]. However, the specific and comparative effects of different anti‐cancer treatments on CRCI remain incompletely understood.

To address these gaps, this study investigated the relationship between anti‐cancer therapies and CRCI, considering objective and subjective cognitive measures as recommended by the International Cancer and Cognition Task Force [23]. Since emotional stress and sleep disturbances associated with a devastating cancer diagnosis can contribute to cognitive problems [7], we compared the change from pre‐to‐post‐therapy cognitive outcomes between different types of cancer therapy. As the results suggested potential effects of chemotherapy on cognition, we further explored the impacts of different chemotherapeutic agents. Finally, since repeated cognitive tests might bias the change scores due to learning effects related to the test procedures, exploratory comparisons were conducted with the cognitive outcomes of cancer‐free women who had repeated measurements under the same test conditions. This helped to contextualise the patients' cognitive changes. Overall, the findings of the CogniFiT study add evidence to inform clinical decision‐making and may support the development of tailored survivorship care plans.

## 2 Methods

### 2.1 Study Population

The CogniFiT study (Cognitive Function in patients with Tumors) is a comparative longitudinal study with three measurement time points: before the start of any systemic or radiotherapy (T0), 7–14 days after the end of that treatment, or, in the case of longer‐lasting endocrine therapy, no later than 6 months after therapy start (T1), and 1 year after T1 (T2). Cognitive function was assessed at T0, T1, and T2. This study recruited women with breast and gynaecological cancer at the National Center for Tumor Diseases Heidelberg between November 2018 and March 2023. Eligible participants were aged 18 years or older, with native German language proficiency, and scheduled to start with radio‐ or systemic therapy. Patients who had tumour surgery, but no further therapy were also enrolled. Exclusion criteria included receipt of any radio‐ or systemic therapy within the past 2 years, known neurodegenerative diseases (such as multiple sclerosis, Alzheimer's disease, or Huntington's disease), psychiatric disorders, neurological damage (e.g., epilepsy or stroke), or current medication that could influence cognitive function.

In parallel to patients' recruitment, cancer‐free women were recruited using advertisements, flyers, and mailing lists. Cancer‐free women served as the comparison group for the patients. The cancer‐free women conducted the cognitive assessments under the same conditions as the cancer patients, with similar timing between assessments as follows: at enrolment (T0), 3 months after T0 (T1), and 1 year after T1 (T2).

### 2.2 Assessment of Cognition

Objective and subjective measures were used to evaluate CRCI. Objective measures included the Hopkins Verbal Learning Test‐Revised (HVLT‐R) [24], Trail Making Test (TMT) [25], Controlled Oral Word Association test (COWA) [26, 27], and Mnemonic Similarity Task (MST) [28, 29].

The HVLT‐R evaluates word‐list learning and verbal memory. Three learning trials and a delayed recall trial were conducted. The number of words correctly recalled by the respondent during three learning trials was tallied as total recall score. The delayed recall trial was administered 20–25 min after the third learning trial without advance notice to participants. Retention was calculated as the percentage of words correctly recalled on the delayed recall trial divided by the higher of the recall scores from the earlier learning trials, multiplied by 100. Higher HVLT‐R scores for total recall (range: 0–36), delayed recall (range: 0–12), and percentage retention (which can exceed 100%) indicate better ability of verbal learning, memory, and retention.

TMT comprises two different parts, Part A (TMT‐A) and Part B (TMT‐B). TMT‐A records the time in seconds required for participants to connect numbers from 1 to 25 on paper in ascending order as quickly and accurately as possible. TMT‐B records the time that participants use to connect 12 numbers and 12 letters in alternating order: 1, A, 2, B, …. TMT‐A represents visual searching and motor speed (typical completion time approximately 20–180 s) while TMT‐B represents executive function related to cognitive flexibility (typical completion time approximately 40–300 s). Shorter completion times indicate better performance.

COWA measures phonemic and semantic fluency [27]. A German version, the Regensburger Wortflüssigkeitstest [26], was used in our study. It contains two tasks. In the phonemic fluency task, participants name as many words as possible within 2 min beginning with a specific letter, either ‘S’, ‘P’, or ‘M’ (typical scores range roughly from 15 to 40 words per letter). In the semantic task, participants name as many words as possible within 60 s from one of the given categories: forenames, animals, and food (typical scores range roughly from 15 to 30 words per category). For both tasks, higher numbers indicate better verbal fluency. At each time point, a different letter and category were given, with the sequence randomly assigned.

The MST consists of two phases. In the first input phase, participants engage in an incidental encoding task, watching pictures of colourful objects that appear on the monitor. Immediately following the second testing phase, participants are required to classify each object on the monitor as ‘New’, ‘Old’, or ‘Similar’. ‘Old’ responses to exact repetitions of images presented in the first input phase (targets) reflect hits, while ‘Old’ responses to new, unseen images (foils) reflect false alarms. ‘Similar’ responses capture images perceptually similar to those from first input phase (lures), and ‘New’ responses indicate correct rejections. From these conditional response probabilities, two key indices are derived: Corrected Recognition Score (REC), which assesses recognition memory for repeated items, representing general recognition memory; and Lure Discrimination Index (LDI), which measures sensitivity to lure items, representing hippocampal‐dependent pattern separation [29].

The MST REC is defined as the probability that an old image is correctly classified as ‘old’ minus the probability that a foil image is incorrectly classified as ‘old’, as shown in Formula (1) [28]:
(1)

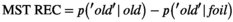




The MST LDI is defined as the probability that a lure image is correctly classified as ‘similar’ minus the probability that a foil image is incorrectly classified as ‘similar’, as shown in Formula (2) [28]:
(2)

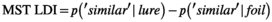




For MST REC, possible range from 0 to 1, and MST LDI, range from −1 to 1; higher values indicate better performance.

Subjective measures included the Functional Assessment of Cancer Therapy—Cognitive scale (FACT‐Cog) [30]: Global Function (GF, range: 0–148), Perceived Cognitive Impairments (PCI, range: 0–80), Perceived Cognitive Abilities (PCA, range: 0–28), impact on Quality of Life (iQoL, range: 0–16), as well as the European Organisation for Research and Treatment of Cancer Quality of Life Questionnaire Core 30 Cognitive Function scale (EORTC QLQ‐C30 CF, range: 0–100) [31]. All subjective measures were coded so that higher scores indicated better functioning.

### 2.3 Assessment of Therapy and Other Factors

Cancer profiles, therapy information, and age were collected from medical records. Weight and height (used to calculate the body mass index [BMI]) were measured by study personnel. Education, current alcohol consumption, and tobacco use were self‐reported at T0 by the participants through questionnaire. Education was categorised based on the highest school or professional degree as follows: academic (university degree), high (high school degree), moderate (middle school or business‐school degree), or basic (any lower education than other categories). Depression was defined by the revised 20‐item Center for Epidemiologic Studies Depression Scale (CESD‐R). Sleep disturbance was evaluated with the 19‐item Pittsburgh Sleep Quality Index (PSQI). Anxiety was measured using the State‐Trait Anxiety Inventory Test (STAI‐T). Social support was assessed with the validated German version of the five‐item ENRICHD Social Support Instrument (ESSI). Details of these measures can be found in our previous article [7].

### 2.4 Statistical Analysis

Linear mixed models were fitted for each objective and subjective cognitive outcome. In order to meet assumptions of normality and homogeneity of variance, right‐skewed data were log‐transformed (base *e*) and left‐skewed data squared. Time was modelled as a discrete categorical factor. Each model included fixed effects for time, therapy group, and their interaction. Age, education, and depression assessed at T0 were included as covariates. A random intercept for participants was specified to account for repeated observations within individuals. Continuous variables were standardised (*z*‐scored) to allow direct comparability of effect sizes. The covariates were selected based on their theoretical association with the dropout mechanism, thereby satisfying the missing at random assumption and ensuring that the linear mixed models handle missing data effectively and provide unbiased parameter estimation within the likelihood‐based framework. Other factors such as tobacco use, alcohol consumption, anxiety, sleep quality, and social support were checked for potential confounding but did not change the results. Results are reported as standardised effect estimates with corresponding 95% confidence intervals (95% CIs) contrasting the changes from pre‐ to post‐treatment between different groups. To further evaluate the contributions of specific chemotherapeutic agents, similar linear mixed models were fitted in which the general therapy variable was replaced with categorical indicators for taxane regimens (docetaxel, paclitaxel, and no taxane), carboplatin (yes and no), and anthracycline (yes and no). Only patients treated with chemotherapy were included in this additional analysis. Cyclophosphamide was not considered as a separate covariate because it was co‐administered with anthracyclines, which would have introduced collinearity into the model. In addition, exploratory comparisons were conducted between patients and cancer‐free women using the same linear mixed model framework, with cancer‐free women as a reference group. Given the exploratory nature of this study, both unadjusted and Benjamini–Hochberg false discovery rate (FDR)‐adjusted *p* values are provided for reference to assess the robustness of the findings to multiplicity. All analyses were two‐sided, exploratory, and conducted in R (version 4.5.0).

## 3 Results

A total of 234 patients from the CogniFiT study with completed baseline assessments and treatment information were included in the final analysis, as shown in the flowchart in Figure 1. In addition, 41 cancer‐free women were also included for explorative analyses. Characteristics at T0 stratified by therapy are summarised in Table 1. Between T0 and T1, 60 patients received chemotherapy, 48 endocrine therapy, 24 radiotherapy, 93 combined endocrine and radiotherapy, and 9 had surgery only. The mean age of all patients was 55.4 years (SD = 11.6), with patients receiving radiotherapy being slightly older (mean 60.8 years) and those on chemotherapy slightly younger (mean 53.1 years). The mean BMI was 26.6 kg/m^2^ (SD = 6.2), with minor differences across therapy groups. Education levels were generally moderate to high across all therapy groups. Psychosocial measures such as depression, anxiety, and sleep disturbances tended to be higher in patients receiving chemotherapy or endocrine therapy compared with those receiving surgery only. Compared to the patients, cancer‐free women were younger (mean age 49.3 years, SD = 9.8), had a comparable mean BMI, a higher proportion held an academic degree, reported lower levels of depression and anxiety, experienced fewer sleeping disturbances, and showed comparable levels of social support, alcohol consumption, and cigarette smoking. Regarding cancer‐related data, the majority of patients were diagnosed with breast cancer (83.8%) or ductal carcinoma in situ (13.2%). Most had no metastases (98.7%), and the largest proportion of tumours (65.0%) were classified as grade 2. Time intervals between T0 and T1 varied among therapy groups due to differences in therapy duration, as shown in Table S1.

**FIGURE 1:**
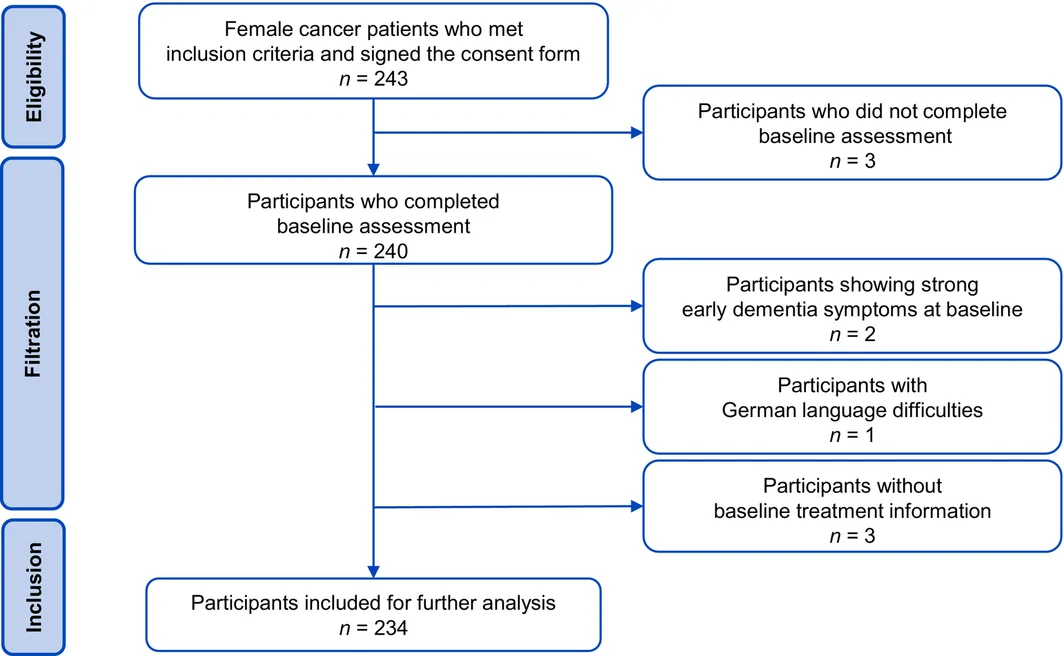
Study flowchart.

**TABLE 1:** Characteristics of patients receiving different anti‐cancer therapies and cancer‐free women at T0.

Characteristics at T0	Chemotherapy (*n* = 60)	Endocrine therapy (*n* = 48)	Radiotherapy (*n* = 24)	Endocrine + radiotherapy (*n* = 93)	Surgery only (*n* = 9)	Total patients (*n* = 234)	Cancer‐free women (*n* = 41)
Age, year, mean (SD)	53.1 (12.5)	56.1 (11.4)	60.8 (14.0)	55.2 (10.0)	55.3 (12.7)	55.4 (11.6)	49.3 (9.8)
BMI, kg/m^2^, mean (SD)	26.6 (6.2)	25.8 (5.4)	26.1 (4.8)	25.3 (5.4)	23.7 (4.7)	25.7 (5.5)	24.5 (4.4)
Education level, *n* (%)							
Basic	4 (6.7)	5 (10.4)	3 (12.5)	12 (12.9)	0	24 (10.3)	1 (2.4)
Moderate	19 (31.7)	18 (37.5)	6 (25.0)	33 (35.5)	2 (22.2)	78 (33.3)	9 (22.0)
High	15 (25.0)	8 (16.7)	6 (25.0)	20 (21.5)	2 (22.2)	51 (21.8)	8 (19.5)
Academic	22 (36.7)	17 (35.4)	9 (37.5)	28 (30.1)	5 (55.6)	81 (34.6)	23 (56.1)
Depression (CESD‐R), median [Q1, Q3]	19.2 [9.6, 33.2]	18.3 [6.3, 36.7]	17.5 [3.0, 31.3]	10.0 [5.0, 25.0]	10.0 [1.7, 27.5]	15.0 [5.0, 31.7]	5.0 [1.7, 11.7]
Anxiety (STAI), median [Q1, Q3]	35.0 [25.0, 48.3]	33.3 [20.4, 45.8]	30.0 [19.2, 50.0]	25.0 [15.0, 41.7]	25.0 [16.7, 40.4]	30.0 [18.3, 45.0]	24.2 [10.4, 35.0]
Sleep disturbances (PSQI), median [Q1, Q3]	8.0 [5.0, 10.0]	8.5 [5.0, 11.0]	7.0 [4.0, 11.0]	5.0 [3.0, 8.0]	4.0 [4.0, 8.0]	6.0 [4.0, 10.0]	4.0 [4.0, 5.0]
Social support (ESSI), median [Q1, Q3]	23.0 [20.1, 25.0]	22.0 [20.0, 24.0]	23.0 [20.3, 24.8]	23.0 [21.0, 25.0]	24.0 [20.0, 25.0]	23.0 [20.0, 25.0]	22.0 [20.0, 24.0]
Alcohol consumption, *n* (%)							
≤ 10 g/day	53 (88.3)	40 (83.3)	19 (79.2)	78 (83.9)	8 (88.9)	198 (84.6)	36 (87.8)
> 10 g/day	7 (11.7)	6 (12.5)	4 (16.7)	14 (15.1)	1 (11.1)	32 (13.7)	5 (12.2)
No information	0	2 (4.2)	1 (4.2)	1 (1.1)	0	4 (1.7)	0
Cigarette smoking, *n* (%)							
Non‐smoker	31 (51.7)	24 (50.0)	8 (33.3)	41 (44.1)	6 (66.7)	110 (47.0)	23 (56.1)
Former smoker	21 (35.0)	18 (37.5)	9 (37.5)	40 (43.0)	2 (22.2)	90 (38.5)	12 (29.3)
Current smoker	8 (13.3)	5 (10.4)	7 (29.2)	12 (12.9)	1 (11.1)	33 (14.1)	6 (14.6)
No information	0	1 (2.1)	0	0	0	1 (0.4)	0
Cancer type, *n* (%)							NA
Breast cancer	52 (86.7)	42 (87.5)	15 (62.5)	83 (89.2)	4 (44.4)	196 (83.8)	
DCIS	1 (1.7)	6 (12.5)	9 (37.5)	10 (10.8)	5 (55.6)	31 (13.2)	
Ovarian	5 (8.3)	0	0	0	0	5 (2.1)	
Endometrium	1 (1.7)	0	0	0	0	1 (0.4)	
Cervix	1 (1.7)	0	0	0	0	1 (0.4)	
Metastasis, *n* (%)							NA
Yes	2 (3.3)	0	0	1 (1.1)	0	3 (1.3)	
No	58 (96.7)	48 (100.0)	24 (100.0)	92 (98.9)	9 (100.0)	231 (98.7)	
Cancer grade, *n* (%)							NA
I	2 (3.3)	8 (16.7)	10 (41.7)	28 (30.1)	1 (11.1)	49 (20.9)	
II	32 (53.3)	39 (81.2)	13 (54.2)	62 (66.7)	6 (66.7)	152 (65.0)	
III	26 (43.3)	1 (2.1)	1 (4.2)	3 (3.2)	2 (22.2)	33 (14.1)	

*Note:* T0, before the start of any systemic therapy or radiotherapy.

Abbreviations: CESD‐R, Center for Epidemiologic Studies Depression Scale–Revised; DCIS, ductal carcinoma in situ; ESSI, ENRICHD Social Support Instrument; NA, not available; PSQI, Pittsburgh Sleep Quality Index; Q1, first quartile; Q3, third quartile; SD, standard deviation.

Table 2 presents the results of the linear mixed models investigating changes in cognitive outcomes from pre‐ to post‐treatment. Compared to patients receiving chemotherapy, those under endocrine therapy showed shorter times completing TMT‐A (*β* = −0.36, 95% CI: −0.69 to −0.03) and reported better subjective cognitive functioning (FACT‐Cog GF: *β* = 0.39, 95% CI: 0.11–0.67; PCI: *β* = 0.47, 95% CI: 0.19–0.75; EORTC QLQ‐C30 CF: *β* = 0.50, 95% CI: 0.18–0.82). Estimates of the other therapy groups tended also in a beneficial direction compared to chemotherapy for TMT‐A and subjective outcomes, but associations were less pronounced and not significant. Other objective measures, including HVLT‐R tests, TMT‐B, COWA, and MST tests, did not show meaningful differences between therapies. After FDR correction, the association between chemotherapy and subjective cognition remained significant, whereas associations with TMT‐A at T1 attenuated. Marginal *R* ^2^ indicated modest variance explained by fixed effects, whereas substantially higher conditional *R* ^2^ values reflected pronounced inter‐individual variability captured by participant‐level random effects. Given these associations with chemotherapy, we further investigated the contribution of specific chemotherapeutic agents on cognitive impairments. Among the 60 patients who received chemotherapy, eight were treated with docetaxel, 49 with paclitaxel, while only three did not receive a taxane. Moreover, 14 patients received carboplatin, 31 were treated with an anthracycline (epirubicin or doxorubicin), and 35 with cyclophosphamide. The most common scheme included four cycles [4×] epirubicin and cyclophosphamide followed by 12× paclitaxel. The remaining 174 patients did not receive any chemotherapeutic agent. The results of the linear mixed models based on 60 patients treated with chemotherapy are summarised in Table S2. A tendency towards poorer MST LDI score was observed with patients treated with anthracycline chemotherapy (*β* = −0.57, 95% CI: −1.13 to −0.01) compared with patients treated with non‐anthracycline chemotherapy. Other agents did not show significant effects.

**TABLE 2:** Differences in changes in objective and subjective cognitive measurements from T0 to T1 between therapy groups.

Dependent variables	Therapies (compared to chemotherapy as reference)													
	Endocrine therapy			Radiotherapy			Endocrine + Radiotherapy			Surgery only			Marginal *R* ^2^	Conditional *R* ^2^
	*β* (95% CI)	*p*	Adj. *p*	*β* (95% CI)	p	Adj. *p*	*β* (95% CI)	*p*	Adj. *p*	*β* (95% CI)	*p*	Adj. *p*		
Objective measures														
HVLT‐R retention^a^	0.09 (−0.42, 0.60)	0.74	0.86	0.38 (−0.28, 1.04)	0.26	0.72	−0.16 (−0.60, 0.28)	0.47	0.77	0.24 (−0.70, 1.18)	0.62	0.81	6%	18%
HVLT‐R total recall^a^	−0.15 (−0.51, 0.21)	0.41	0.77	0.32 (−0.14, 0.79)	0.17	0.63	−0.08 (−0.39, 0.23)	0.61	0.81	−0.08 (−0.75, 0.58)	0.80	0.88	16%	61%
HVLT‐R delayed recall^a^	0.04 (−0.34, 0.42)	0.83	0.88	0.34 (−0.16, 0.84)	0.18	0.63	0.04 (−0.28, 0.37)	0.80	0.88	0.13 (−0.57, 0.84)	0.71	0.86	16%	55%
Time to complete TMT‐A^b^	**−0.36 (−0.69, −0.03)** *	**0.03**	0.30	−0.12 (−0.55, 0.31)	0.58	0.81	−0.15 (−0.43, 0.14)	0.32	0.73	−0.45 (−1.08, 0.17)	0.15	0.63	28%	65%
Time to complete TMT‐B^b^	−0.12 (−0.41, 0.18)	0.43	0.77	0.13 (−0.25, 0.51)	0.51	0.77	0.06 (−0.19, 0.31)	0.64	0.81	0.01 (−0.55, 0.56)	0.97	0.99	31%	73%
COWA phonemic^a^	0.17 (−0.29, 0.63)	0.46	0.77	0.37 (−0.22, 0.96)	0.22	0.67	0.16 (−0.24, 0.56)	0.42	0.77	0.42 (−0.39, 1.23)	0.31	0.73	7%	42%
COWA semantic^a^	0.05 (−0.39, 0.50)	0.82	0.88	−0.28 (−0.86, 0.29)	0.33	0.73	0.11 (−0.28, 0.49)	0.59	0.81	−0.36 (−1.15, 0.42)	0.37	0.73	10%	45%
MST REC^a^	−0.03 (−0.49, 0.43)	0.89	0.91	−0.09 (−0.68, 0.49)	0.75	0.86	−0.10 (−0.50, 0.30)	0.63	0.81	0.68 (−0.15, 1.52)	0.11	0.63	1%	37%
MST LDI^a^	−0.27 (−0.61, 0.06)	0.10	0.63	−0.13 (−0.55, 0.30)	0.56	0.81	0.02 (−0.27, 0.31)	0.88	0.91	0.43 (−0.17, 1.04)	0.16	0.63	24%	68%
Subjective measures														
FACT‐Cog GF^a^	**0.39 (0.11, 0.67)** *	**0.01**	**0.07**	0.20 (−0.16, 0.56)	0.28	0.73	0.12 (−0.12, 0.36)	0.34	0.73	0.38 (−0.13, 0.89)	0.15	0.63	39%	74%
FACT‐Cog PCI^a^	**0.47 (0.19, 0.75)** *	**< 0.01**	**0.04**	0.24 (−0.12, 0.60)	0.19	0.63	0.21 (−0.04, 0.45)	0.10	0.63	0.45 (−0.07, 0.96)	0.09	0.63	35%	74%
FACT‐Cog PCA^a^	0.15 (−0.15, 0.46)	0.33	0.73	0.08 (−0.31, 0.47)	0.70	0.56	0.11 (−0.15, 0.37)	0.41	0.77	0.44 (−0.12, 0.99)	0.12	0.63	31%	70%
FACT‐Cog iQoL^a^	0.12 (−0.23, 0.47)	0.50	0.77	0.20 (−0.24, 0.65)	0.37	0.73	−0.08 (−0.38, 0.22)	0.60	0.81	−0.07 (−0.70, 0.56)	0.83	0.88	38%	60%
EORTC QLQ‐C30 CF^a^	**0.50 (0.18, 0.82)** *	**< 0.01**	**0.05**	0.22 (−0.18, 0.63)	0.28	0.73	0.14 (−0.13, 0.41)	0.30	0.73	−0.10 (−0.67, 0.47)	0.74	0.86	36%	68%

*Note:* Separate linear mixed models were computed for each cognitive measure with therapy as the independent variable, mandatorily adjusted for age, education, and depression at T0. T0, before the start of any systemic or radiotherapy; T1, 7–14 days after the end of that treatment, or, in the case of longer‐lasting endocrine therapy, no later than 6 months after therapy start.

Abbreviations: *β*, standardised regression coefficient; Adj. *p*, *p* value adjusted for multiple comparisons using the Benjamini–Hochberg false discovery rate procedure; CI, confidence interval; Conditional R^2^, proportion of variance explained by both fixed and random effects; COWA, Controlled Oral Word Association Test; EORTC QLQ‐C30 CF, European Organisation for Research and Treatment of Cancer Quality of Life Questionnaire Core 30 Cognitive Function scale; FACT‐Cog GF, Functional Assessment of Cancer Therapy–Cognitive Function Global Function; FACT‐Cog iQoL, Functional Assessment of Cancer Therapy–Cognitive Function Impact on Quality of Life; FACT‐Cog PCA, Functional Assessment of Cancer Therapy–Cognitive Function Perceived Cognitive Abilities; FACT‐Cog PCI, Functional Assessment of Cancer Therapy–Cognitive Function Perceived Cognitive Impairments; HVLT‐R, Hopkins Verbal Learning Test–Revised; Marginal *R* ^2^, proportion of variance explained by fixed effects only; MST LDI, Mnemonic Similarity Task Lure Discrimination Index; MST REC, Mnemonic Similarity Task Corrected Recognition Score; *p*, two‐sided unadjusted *p* value; TMT‐A/B, Trail Making Test Part A/Part B.

* *p* < 0.05 is shown in bold.

^a^ Higher values indicate better cognitive function; therefore, a positive *β* indicates better cognitive status.

^b^ Lower values indicate better cognitive function; therefore, a negative *β* indicates better cognitive status.

To explore whether the cognitive changes associated with different therapies persisted over time, contrasts regarding the follow‐up outcomes at T2 derived from the linear mixed models are presented in Table S3. Regarding the objective measures at T2, endocrine therapy was associated with lower performance on HVLT‐R total recall (*β* = −0.40, 95% CI: −0.76 to −0.03) compared to chemotherapy, suggesting persistent deficits in verbal learning and memory 1 year later, and higher perceived cognitive impairments (FACT‐Cog PCI: *β* = 0.34, 95% CI: 0.05–0.63). Compared with the chemotherapy group, participants who received radiotherapy (*β* = 0.42, 95% CI: 0.03–0.82) or combined endocrine plus radiotherapy (*β* = 0.25, 95% CI: 0.00–0.51) demonstrated poorer TMT‐B performance, reflected by higher standardised log‐transformed completion times. However, these associations did not remain statistically significant after adjustment for multiple comparisons.

Figure 2 presents similar standardised estimates (95% CIs) of group‐differences in changes of cognitive performance at T1 and T2 relative to T0 as presented above, derived from the same linear mixed models, but now contrasting with the group of cancer‐free women. Results of the surgery‐only group are omitted due to very small sample size and limited space. Regarding the objective measures, endocrine therapy (without or with radiotherapy) showed associations with poorer verbal abilities (HVLT‐R total and/or delayed recall) compared to cancer‐free women at T1 but particularly at T2. Subjective measures indicated poorer perceived cognitive function compared to cancer‐free women regarding changes at T1 among patients, which were most pronounced among those receiving chemotherapy, followed by those with combined endocrine plus radiotherapy. Regarding changes at T2, subjectively perceived cognitive complaints of patients who had received chemotherapy, compared to cancer‐free women, seemed to be less pronounced than at T1.

**FIGURE 2:**
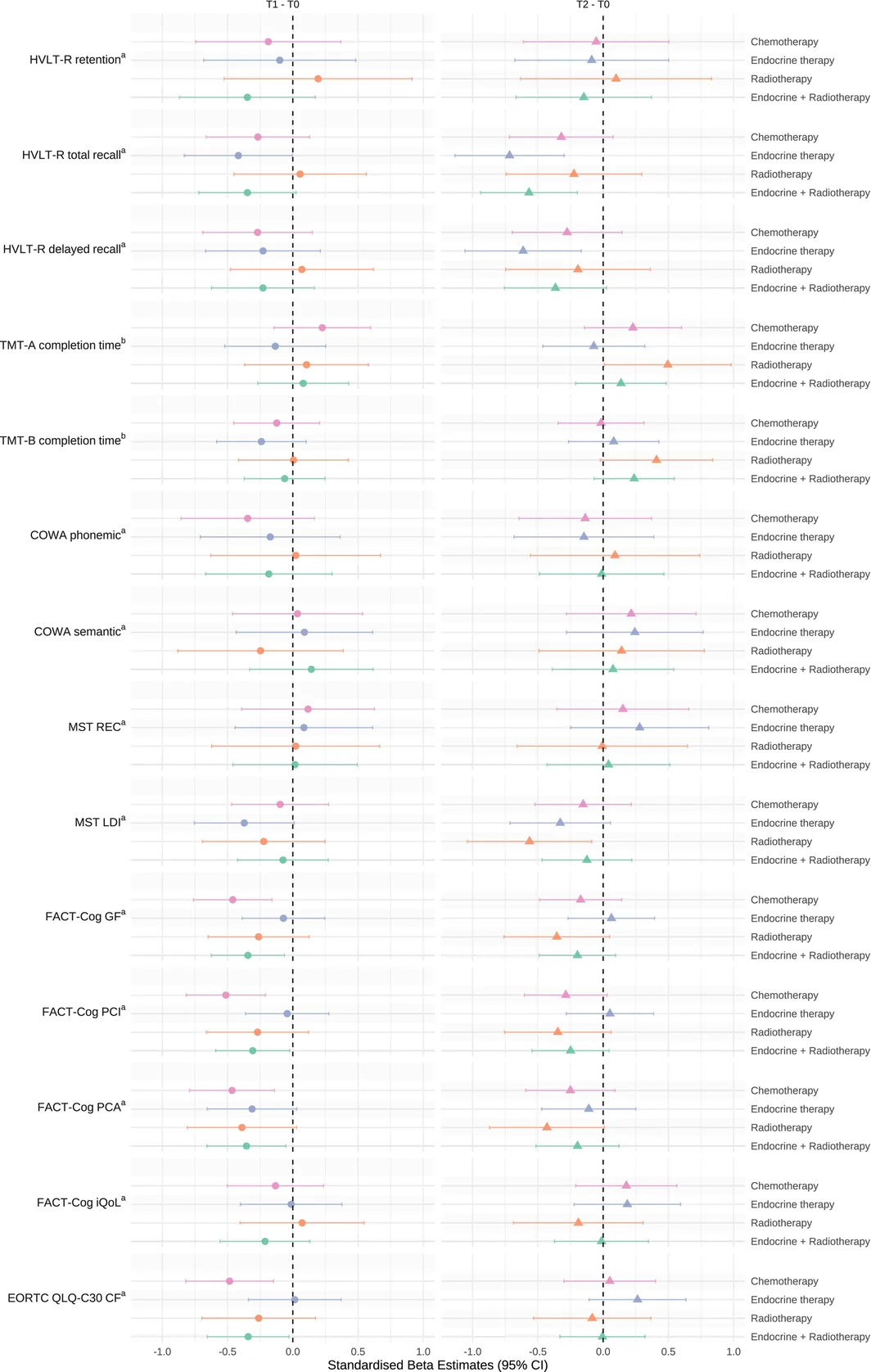
Differences in cognitive measures at T1–T0 and T2–T0 between anti‐cancer therapies and cancer‐free women. Separate mixed models were computed for each cognitive measure, with therapy as the independent variable, mandatorily adjusted for baseline age, education, and depression. The group “Surgery only” is not shown in this figure due to limited interpretability and constraints of figure length. T0, before the start of any systemic therapy or radiotherapy; T1, 7–14 days after the end of that treatment, or in the case of longer‐lasting endocrine therapy, no later than 6 months after therapy start; T2, 1 year after the end of that treatment. ^a^Higher values indicate better cognitive function; thus, a positive *β* indicates better cognitive status. ^b^Lower values indicate better cognitive function; thus, a negative *β* indicates better cognitive status. COWA, Controlled Oral Word Association Test; EORTC QLQ‐C30 CF, European Organisation for Research and Treatment of Cancer Quality of Life Questionnaire Core 30, Cognitive Function scale; FACT‐Cog GF, Functional Assessment of Cancer Therapy–Cognitive Function Global Function; FACT‐Cog iQoL, Functional Assessment of Cancer Therapy–Cognitive Function Impact on Quality of Life; FACT‐Cog PCA, Functional Assessment of Cancer Therapy–Cognitive Function Perceived Cognitive Abilities; FACT‐Cog PCI, Functional Assessment of Cancer Therapy–Cognitive Function Perceived Cognitive Impairments; HVLT‐R, Hopkins Verbal Learning Test–Revised; MST LDI, Mnemonic Similarity Task Lure Discrimination Index; MST REC, Mnemonic Similarity Task Corrected Recognition Score; TMT‐A/B, Trail Making Test Part A/Part B.

## 4 Discussion

Our results indicate that anti‐cancer therapies may be associated with changes in cognitive function. Patients with breast or gynaecological cancer reported perceived cognitive impairments after chemotherapy, where objective impairments in visual searching and motor speed were also evident. While cognitive difficulties after chemotherapy seemed to diminish with time, the commonly longer‐term ongoing endocrine therapy was associated with continued impairments in memory. Radiotherapy showed no clear immediate cognitive effects but may negatively affect executive function 1 year later. An indication of possible anthracycline effects on certain cognitive functions merits further investigation in future studies.

The finding that chemotherapy is associated with worse cognitive function shortly after treatment supports the growing evidence for the acute cognitive effect of chemotherapy. This aligns with a meta‐analysis of longitudinal cohorts reporting that chemotherapy is linked to subjective cognitive impairment immediately after treatment completion [32]. Our results are consistent with those of the Trial Assigning Individualized Treatment Options (TAILORx), in which women with early breast cancer were randomly assigned to chemotherapy followed by endocrine therapy or endocrine therapy alone [33]. FACT‐Cog PCI was used as the primary endpoint to assess CRCI at baseline and at 3, 6, 12, 24, and 36 months in that trial. Women randomly assigned to chemotherapy followed by endocrine therapy reported significantly greater cognitive impairment from baseline to 3 and 6 months compared with women assigned to endocrine therapy alone. At 12, 24, and 36 months, PCI scores were similar between both groups. These studies are in line with our study, suggesting an acute effect of chemotherapy on perceived cognitive impairments and that the effect might abate over time. Our study contributes additional evidence by identifying impairments in objective cognition performance, providing information beyond the subjective measures reported in the TAILORx study.

Importantly, our data suggests that the chemotherapy‐related cognitive changes may be temporary or at least diminish over time. A previous review using functional neuroimaging as a tool for CRCI diagnosis similarly suggested that brain abnormalities may begin early in the disease course, peak shortly after chemotherapy, and partially recover over time [34]. A meta‐analysis of 11 longitudinal studies found significant chemotherapy‐induced impairments in attention and concentration among breast cancer patients [35], which aligns with our findings, particularly for TMT‐A test performance.

Clinical studies and systematic reviews have shown that various chemotherapeutic agents differ in their neurotoxic profiles [36, 37]. While most research has focused on composite chemotherapy exposure rather than isolated drugs, our findings further suggest that the effects of chemotherapy on cognition might vary depending on the specific neurotoxic profiles of the chemotherapeutic agents administered. In our study, anthracycline was related to lower MST LDI compared to non‐anthracycline chemotherapy regimens, suggesting worse hippocampal‐dependent pattern separation ability, but not significant on verbal memory (HVLT‐R). Evidence regarding verbal memory is less consistent. A cross‐sectional study of 62 primary breast cancer survivors who were more than 2 years off‐therapy reported greater impairments in HVLT‐R verbal memory among patients treated with anthracycline‐based compared with non‐anthracycline‐based chemotherapy [38]. In contrast, a longitudinal study reported no significant association between neurocognitive function tests and anthracycline‐containing chemotherapy based on repeatedly measured of verbal learning (California Verbal learning Test), processing speed (TMT‐A), and executive function (TMT‐B), which is more consistent with our null findings for verbal memory [39]. While a previous study provided some evidence that carboplatin‐based chemotherapy could cause CRCI accompanied by functional changes among non‐small cell lung cancer patients [40], our study did not show significant associations of carboplatin with cognitive outcomes compared to non‐carboplatin chemotherapy regimens. These findings highlight that the impact of chemotherapy on cognition is not uniform and may depend on the neurotoxic profiles of the individual agents. However, the sample sizes of the chemotherapy subgroups were small, and the association of anthracycline and hippocampal‐dependent pattern separation (MST LDI) likewise lost statistical significance after adjustment for multiple comparisons. Hence, results must be interpreted with caution, but merit further investigation in future studies due to their clinical relevance.

Endocrine therapy typically is administered for a year or longer, thus might have been ongoing between T1 and T2. At the one‐year follow‐up assessment (T2), patients receiving endocrine therapy (with or without radiotherapy) exhibited poorer verbal learning and memory performance (HVLT‐R total and delayed recall) compared with those receiving chemotherapy. Recent evidence further suggests a potential exposure‐dependent association between tamoxifen treatment and the cognitive domains of verbal learning, processing speed, executive function, and motor functioning [41]. Proposed mechanisms underlying CRCI associated with endocrine therapy include the effects of both aromatase inhibitors and selective oestrogen receptor modulators on cognitive changes involving verbal learning and memory [2, 42]. Their distinct mechanisms of altering oestrogen signalling and effects on hippocampal and frontal brain function may contribute to differential cognitive outcomes, which warrant further investigation.

Our study does not identify clear effects of radiotherapy of the breast on cognitive performance directly after the end of radiotherapy but suggests a potential negative influence on executive function related to cognitive flexibility (TMT‐B) 1 year after radiotherapy‐containing therapies. Similarly, a longitudinal study that followed breast cancer patients for 1 year after cancer treatments also found that radiotherapy was associated with altered local functional connectivity and reduced white matter integrity [43]. Viewed together, these findings raise the possibility that radiotherapy‐related effects may emerge over time and contribute to subtle long‐term cognitive changes. However, further research is needed to confirm this hypothesis.

This study has several limitations. First, although we applied a broad spectrum of objective and subjective cognitive assessment, as recommended by the International Cancer and Cognition Task Force [23], additional or alternative methods may have detected CRCI more precisely. Standard neuropsychological tests, while widely used, may not capture the subtle cognitive changes associated with CRCI [44]. More sensitive approaches, such as neurophysiological assessments [45] or brain‐based neuroimaging techniques [46] can provide more precise indicators for mechanistic insight and clinical intervention. As a result, our findings may underestimate the extent of cognitive alterations, particularly at early or mild stages. This limitation reflects not a flaw of our approach, but an inherent constraint of using neuropsychological tests only. Nonetheless, more advanced assessments can be difficult to interpret at the individual level, which limits their feasibility in routine clinical research, even though they provide important mechanistic insights into CRCI [45]. Second, since the intervals between T0 and T1 differed depending on the therapy, different learning effects on repeated objective cognitive tests cannot be excluded. However, the observed therapy‐related changes appear not to be related to therapy‐specific interval length. Finally, the characteristics of treatment groups differed due to non‐randomisation, and specific patient characteristics likely influenced the assignment of the therapies. Thus, despite comprehensive adjustments, residual confounding cannot be ruled out. Generally, exploratory analyses provide valuable information with both raw and adjusted *p* values, but this needs further confirmation.

## 5 Conclusions

Taken together, our findings highlight potential cognitive impacts of anti‐cancer therapies and provide valuable guidance for clinical decision‐making, personalised care, and patient counselling. Patients should be informed that cognitive impairments may occur, especially during certain types of chemotherapy, endocrine therapy, and radiotherapy. However, those receiving chemotherapy can be reassured that cognitive difficulties often improve after treatment completion. Additionally, there remains a continued need to establish evidence‐based interventions that both mitigate CRCI and strengthen cancer survivors' capacity to cope.

## Author Contributions

**Qiqi Lei:** writing – original draft, formal analysis. **Martina E. Schmidt:** writing – review and editing, formal analysis, supervision, data curation. **Philipp Zimmer:** conceptualization, writing – review and editing, formal analysis. **Patric Meyer:** writing – review and editing, methodology. **Andreas Schneeweiss:** writing – review and editing, resources. **Karen Steindorf:** conceptualization, writing – review and editing, supervision, formal analysis, project administration.

## Ethics Statement

This study was conducted in accordance with the ethical standards of the Helsinki Declaration. The study was approved by the Ethic Committee of the Medical Faculty of the University of Heidelberg (approval number S‐489/2018). All patients have given written informed consent. The study was registered at the German clinical trial registry (DRKS00015757, Deutsches Register Klinischer Studien).

## Conflicts of Interest

The authors declare no conflicts of interest.

## Supporting information


**Table S1:** Time intervals from T0 to T1.
**Table S2:** Differences in changes in objective and subjective cognitive measurements from T0 to T1 between chemotherapeutic agents among patients receiving chemotherapy.
**Table S3:** Differences in changes in objective and subjective cognitive measurements from T0 to T2 between therapy groups.

## Data Availability

The data used in this study is based on the CogniFiT study. Data access can be granted upon formal request for scientific cooperation. Further data and information that support the findings of this study are available from the corresponding author upon reasonable request.
