# Supplementary material for: Assessment of Cancer‐Related Cognitive Impairments Across Anti‐Cancer Therapies in a Predominantly Breast Cancer Cohort
Source: Int J Cancer. 2026 Aug 7;160(1):85–97. doi: 10.1002/ijc.70691 (PMC13729997; doi:10.1002/ijc.70691)
Supplement: Supplementary file 1 [file IJC-160-85-s001.pdf]

Assessment of cancer-related cognitive impairments across anti-cancer therapies in a predominantly breast cancer cohort

Qiqi Lei, Martina E. Schmidt, Philipp Zimmer, Patric Meyer, Andreas Schneeweiss, Karen Steindorf

Table of Contents

Supplementary Tables 2

Supplementary Table 1. Time intervals from T0 to T1 2

Supplementary Table 2. Differences in changes in objective and subjective cognitive measurements from T0 to T1 between chemotherapeutic agents among patients receiving chemotherapy† 3

Supplementary Table 3. Differences in changes in objective and subjective cognitive measurements from T0 to T2 between therapy groups† 4

Supplementary Tables

Supplementary Table 1. Time intervals from T0 to T1

Therapy	Time interval from T0 to T1, days, median [Q1, Q3]
Chemotherapy	160 [139, 182]
Endocrine therapy	100 [91, 125]
Endocrine and radiotherapy	61 [53, 73]
Radiotherapy	57 [45, 69]
Surgery only	112 [100, 123]

Notes: T0, before the start of any systemic or radiotherapy; T1, 7-14 days after the end of that treatment, or, in the case of longer-lasting endocrine therapy, no later than six months after therapy start; Q1, first quartile; Q3, third quartile.

Supplementary Table 2. Differences in changes in objective and subjective cognitive measurements from T0 to T1 between chemotherapeutic agents among patients receiving chemotherapy†

Dependent variables	Chemotherapeutic agents											
	**Docetaxel vs. Non-taxane**			**Paclitaxel vs. Non-taxane**			**Carboplatin vs. Non-carboplatin**			**Anthracycline vs. Non-anthracycline**		
	β (95% CI)	*P*	Adj. *P*	β (95% CI)	*P*	Adj. *P*	β (95% CI)	*P*	Adj. *P*	β (95% CI)	*P*	Adj. *P*
**Objective measures**												
HVLT-R retention[a]	1.10 (-0.88, 3.08)	0.27	0.82	1.37 (-0.49, 3.23)	0.15	0.82	-0.51 (-1.33, 0.31)	0.22	0.82	0.14 (-0.61, 0.89)	0.71	0.92
HVLT-R total recall[a]	0.32 (-1.21, 1.86)	0.68	0.92	0.14 (-1.30, 1.57)	0.85	0.92	-0.08 (-0.72, 0.56)	0.81	0.92	0.17 (-0.41, 0.75)	0.56	0.86
HVLT-R delayed recall[a]	0.68 (-0.88, 2.24)	0.39	0.82	0.76 (-0.70, 2.22)	0.30	0.82	-0.26 (-0.91, 0.39)	0.43	0.82	0.45 (-0.14, 1.04)	0.14	0.82
Time to complete TMT-A[b]	0.01 (-1.24, 1.26)	0.99	0.99	-0.59 (-1.76, 0.58)	0.32	0.82	-0.49 (-1.01, 0.03)	0.07	0.82	-0.00 (-0.48, 0.47)	0.99	0.99
Time to complete TMT-B[b]	-0.52 (-1.67, 0.64)	0.38	0.82	-0.67 (-1.76, 0.41)	0.22	0.82	0.05 (-0.43, 0.54)	0.83	0.92	0.13 (-0.31, 0.57)	0.57	0.86
COWA phonemic[a]	-1.53 (-3.45, 0.38)	0.12	0.82	-0.76 (-2.60, 1.09)	0.42	0.82	0.27 (-0.42, 0.95)	0.44	0.82	0.08 (-0.54, 0.69)	0.80	0.92
COWA semantic[a]	-1.07 (-3.18, 1.03)	0.31	0.82	-0.95 (-2.98, 1.09)	0.36	0.82	-0.36 (-1.12, 0.39)	0.34	0.82	0.20 (-0.48, 0.88)	0.55	0.86
MST REC[a]	0.60 (-1.22, 2.43)	0.52	0.86	1.41 (-0.30, 3.13)	0.11	0.82	0.60 (-0.17, 1.38)	0.13	0.82	-0.07 (-0.77, 0.63)	0.85	0.92
MST LDI[a]	-1.14 (-2.59, 0.31)	0.12	0.82	-0.15 (-1.52, 1.21)	0.82	0.92	-0.15 (-0.77, 0.47)	0.62	0.91	**-0.57 (-1.13, -0.01)**	**0.05**	0.82
**Subjective measures**												
FACT-Cog GF[a]	0.49 (-0.87, 1.85)	0.48	0.86	0.01 (-1.26, 1.29)	0.98	0.99	0.22 (-0.33, 0.77)	0.43	0.82	0.17 (-0.33, 0.67)	0.50	0.86
FACT-Cog PCI[a]	0.24 (-1.15, 1.62)	0.74	0.92	-0.18 (-1.47, 1.12)	0.79	0.92	0.09 (-0.47, 0.65)	0.75	0.92	0.12 (-0.39, 0.63)	0.63	0.92
FACT-Cog PCA[a]	0.62 (-0.83, 2.07)	0.40	0.82	0.08 (-1.28, 1.44)	0.91	0.96	0.07 (-0.51, 0.66)	0.81	0.92	0.27 (-0.26, 0.81)	0.31	0.82
FACT-Cog iQoL[a]	0.99 (-0.67, 2.65)	0.24	0.82	0.46 (-1.10, 2.01)	0.56	0.86	0.57 (-0.10, 1.24)	0.10	0.82	0.26 (-0.35, 0.87)	0.41	0.82
EORTC QLQ-C30 CF[a]	0.26 (-1.18, 1.69)	0.73	0.92	0.56 (-0.80, 1.92)	0.42	0.82	0.29 (-0.31, 0.88)	0.34	0.82	-0.23 (-0.77, 0.30)	0.39	0.82

Notes: T0, before the start of any systemic or radiotherapy; **T1**, 7-14 days after the end of that treatment, or, in the case of longer-lasting endocrine therapy, no later than six months after therapy start;
†, Separate linear mixed models were computed for each cognitive measure with chemotherapeutic agents as the independent variable, mandatorily adjusted for age, education, and depression at T0;
β, standardized regression coefficient;
CI, confidence interval;
P, two-sided unadjusted P value;
Adj. *P*, *P* value adjusted for multiple comparisons using the Benjamini–Hochberg false discovery rate procedure;
a, higher values indicate better cognitive function; therefore, a positive β indicates better cognitive status;
b, lower values indicate better cognitive function; therefore, a negative β indicates better cognitive status;
COWA, Controlled Oral Word Association Test; **EORTC QLQ-C30 CF**, European Organisation for Research and Treatment of Cancer Quality of Life Questionnaire Core 30 Cognitive Function scale; **FACT-Cog GF**, Functional Assessment of Cancer Therapy–Cognitive Function Global Function; **FACT-Cog iQoL**, Functional Assessment of Cancer Therapy–Cognitive Function Impact on Quality of Life; **FACT-Cog PCA**, Functional Assessment of Cancer Therapy–Cognitive Function Perceived Cognitive Abilities; **FACT-Cog PCI**, Functional Assessment of Cancer Therapy–Cognitive Function Perceived Cognitive Impairments; **HVLT-R**, Hopkins Verbal Learning Test–Revised; MST LDI, Mnemonic Similarity Task Lure Discrimination Index; **MST REC**, Mnemonic Similarity Task Corrected Recognition Score; **TMT-A/B**, Trail Making Test Part A/Part B.

Supplementary Table 3. Differences in changes in objective and subjective cognitive measurements from T0 to T2 between therapy groups[†]

Dependent variables	Therapies (compared to chemotherapy as reference)											
	Endocrine therapy			Radiotherapy			Endocrine + Radiotherapy			Surgery only		
	β (95% CI)	*P*	Adj. *P*	β (95% CI)	*P*	Adj. *P*	β (95% CI)	*P*	Adj. *P*	β (95% CI)	*P*	Adj. *P*
**Objective measures**												
HVLT-R retention[a]	-0.03 (-0.55, 0.48)	0.90	0.96	0.15 (-0.52, 0.83)	0.66	0.92	-0.09 (-0.53, 0.34)	0.67	0.92	-0.18 (-1.13, 0.76)	0.70	0.92
HVLT-R total recall[a]	**-0.40 (-0.76, -0.03)***	**0.03**	0.57	0.10 (-0.38, 0.57)	0.69	0.92	-0.25 (-0.55, 0.06)	0.11	0.57	-0.11 (-0.77, 0.56)	0.75	0.92
HVLT-R delayed recall[a]	-0.34 (-0.72, 0.05)	0.09	0.57	0.08 (-0.42, 0.59)	0.75	0.92	-0.09 (-0.41, 0.24)	0.59	0.92	-0.42 (-1.12, 0.29)	0.24	0.74
Time to complete TMT-A[b]	-0.30 (-0.64, 0.04)	0.08	0.57	0.27 (-0.18, 0.71)	0.24	0.74	-0.09 (-0.38, 0.19)	0.53	0.92	-0.31 (-0.94, 0.31)	0.32	0.83
Time to complete TMT-B[b]	0.10 (-0.20, 0.40)	0.53	0.92	**0.42 (0.03, 0.82)***	**0.03**	0.57	**0.25 (0.00, 0.51)***	**0.05**	0.57	0.08 (-0.47, 0.63)	0.78	0.92
COWA phonemic[a]	-0.01 (-0.47, 0.45)	0.97	0.98	0.23 (-0.36, 0.82)	0.45	0.89	0.13 (-0.26, 0.52)	0.52	0.92	-0.45 (-1.26, 0.36)	0.27	0.79
COWA semantic[a]	0.03 (-0.42, 0.48)	0.90	0.96	-0.07 (-0.65, 0.50)	0.80	0.93	-0.14 (-0.52, 0.24)	0.47	0.92	0.10 (-0.69, 0.88)	0.81	0.9.3
MST REC[a]	0.13 (-0.33, 0.59)	0.59	0.92	-0.16 (-0.76, 0.44)	0.61	0.92	-0.11 (-0.50, 0.28)	0.58	0.93	0.69 (-0.14, 1.53)	0.10	0.57
MST LDI[a]	-0.18 (-0.51, 0.16)	0.30	0.82	-0.41 (-0.85, 0.03)	0.07	0.57	0.03 (-0.26, 0.31)	0.84	0.93	0.45 (-0.15, 1.06)	0.14	0.66
**Subjective measures**												
FACT-Cog GF[a]	0.24 (-0.05, 0.52)	0.11	0.57	-0.18 (-0.55, 0.19)	0.33	0.83	-0.02 (-0.27, 0.22)	0.84	0.93	0.35 (-0.18, 0.88)	0.20	0.74
FACT-Cog PCI[a]	**0.34 (0.05, 0.63)***	**0.02**	0.57	-0.06 (-0.44, 0.31)	0.75	0.92	0.04 (-0.21, 0.28)	0.77	0.92	0.51 (-0.03, 1.04)	0.06	0.57
FACT-Cog PCA[a]	0.14 (-0.17, 0.46)	0.38	0.84	-0.18 (-0.58, 0.22)	0.38	0.84	0.05 (-0.21, 0.32)	0.69	0.92	0.25 (-0.33, 0.83)	0.39	0.84
FACT-Cog iQoL[a]	0.01 (-0.35, 0.37)	0.97	0.98	-0.37 (-0.82, 0.09)	0.12	0.57	-0.19 (-0.49, 0.11)	0.22	0.74	-0.00 (-0.66, 0.65)	0.99	0.99
EORTC QLQ-C30 CF[a]	0.21 (-0.11, 0.54)	0.20	0.74	-0.13 (-0.55, 0.28)	0.52	0.92	-0.06 (-0.33, 0.22)	0.69	0.92	0.08 (-0.51, 0.68)	0.78	0.92

Notes: T0, before the start of any systemic or radiotherapy; **T2**, 1 year after the end of that treatment;

†, Separate linear mixed models were computed for each cognitive measure with therapy as the independent variable, mandatorily adjusted for age, education, and depression at T0;

*****, $P < 0.05$;

β, standardized regression coefficient;

CI, confidence interval;

P, two-sided unadjusted P value;

Adj. *P*, *P* value adjusted for multiple comparisons using the Benjamini–Hochberg false discovery rate procedure;

a, higher values indicate better cognitive function; therefore, a positive β indicates better cognitive status;

b, lower values indicate better cognitive function; therefore, a negative β indicates better cognitive status;

COWA, Controlled Oral Word Association Test; **EORTC QLQ-C30 CF**, European Organisation for Research and Treatment of Cancer Quality of Life Questionnaire Core 30 Cognitive Function scale; **FACT-Cog GF**, Functional Assessment of Cancer Therapy–Cognitive Function Global Function; **FACT-Cog iQoL**, Functional Assessment of Cancer Therapy–Cognitive Function Impact on Quality of Life; **FACT-Cog PCA**, Functional Assessment of Cancer Therapy–Cognitive Function Perceived Cognitive Abilities; **FACT-Cog PCI**, Functional Assessment of Cancer Therapy–Cognitive Function Perceived Cognitive Impairments; **HVLT-R**, Hopkins Verbal Learning Test–Revised; **MST LDI**, Mnemonic Similarity Task Lure Discrimination Index; **MST REC**, Mnemonic Similarity Task Corrected Recognition Score; **TMT-A/B**, Trail Making Test Part A/Part B.
